# CTLA-4 immunotherapy exposes differences in immune response along with different tumor progression in colorectal cancer

**DOI:** 10.18632/aging.103765

**Published:** 2020-08-15

**Authors:** Xiaocong Fu, Hua Luo, Yuhui Zheng, Shengpeng Wang, Zhangfeng Zhong, Yitao Wang, Yeguo Yang

**Affiliations:** 1 Institute of Chinese Medical Sciences, State Key Laboratory of Quality Research in Chinese Medicine, University of Macau, Macao, China; 2 Equal contribution

**Keywords:** CTLA-4, effector memory T cell (TEM), central memory T cell (TCM), tumor-immune microenvironment

## Abstract

Tumor growth is accompanied by a changing tumor microenvironment and mutations that increase the resistance to therapy. Here, we used syngeneic models to evaluate the drug response of tumors of the same type of different sizes. We used the *in vivo* efficacy and Ki-67 immunohistochemistry (IHC) assay to assess the difference in responses in response to treatment with the same concentration of anti-CTLA-4. Flow cytometry analysis revealed changes in the immune subpopulations changes the spleen, peripheral blood, lymph node, and tumor tissue across different tumor growth phases. For example, naive CD4+T, CD4+TCM, CD8+TEM, T, B, Treg, CD8+TCM exhibited different percentages depending on the specific immune organ. To further expose the changes in the immune microenvironment, the level of expression of PD-1 and CTLA-4 showed statistically significant difference in related subsets for each four immune tissues in different tumor sizes. In addition, the ratios of CD4 + Teff/ CD4 + Treg and CD8 + T/Treg in corresponding immune tissue were also associated with statistically significant differences alongside tumor growth in different animal models. These results reveal the ongoing changes in the immune microenvironment during tumor progression and anti-CTLA-4 antibody immunotherapy effect depends on the expression level of immune factors.

## INTRODUCTION

Cancer is becoming a worldwide concern. One of the difficulties of treating cancer is that progressive mutations accompany many tumors. This is a huge concern in the case of colorectal cancer (CRC) [1]. The CT26 and Colon26 murine colon tumor syngeneic models have been widely used in cancer-related research, including studies to address the essence of the tumor progression and the tumor microenvironment [2]. Both the models were used in the establishment of cell-line derived xenografts (CDXs) of wild-type colon cancer models in immunocompetent mice [2, 3]. In addition, the tumor models (CT26 and Colon26) have become popular as research tools to address the tumor progression changes concerning immunophenotyping and the relative immune microenvironment, and it was found that the wild-type tumor can be highly immunogenic [4].

The general evaluation of immune function is based on the monitoring of tumor-infiltrating lymphocytes (TILs) and the spleen, lymph node, and blood tissue subpopulations in the immunized animal bearing the tumor cell line [5]. As for the checkpoint factors, it is known that Cytotoxic T-lymphocyte-associated antigen 4 (CTLA-4) plays a crucial role in the regulation of T-cell activation [6–8]. Programmed cell death protein 1 (PD-1)—known as cluster of differentiation 279 (CD279)—is also an important checkpoint in regulating the relative immune response that has become a universal topic in the field of immunotherapy [9, 10]. Given their primary immune function, the monitoring of T-cell populations like CD4 + T, CD8 + T, and the Treg cells and their respective immune function is essential to evaluate the ongoing changes in the tumor microenvironment. Likewise, memory T cells, effector T cells, and Naive T cells also influence progression changes during tumor growth [9, 11, 12].

The CT26 and Colon26 syngeneic models have been used in immunocompetent recipients to evaluate the dynamics of the immune subpopulations during tumor growth. It has already been established that T-cell subsets are the main subpopulations that are associated with tumor progression in many cancer patients. Treg cells can suppress antitumor immune responses and establish immunosuppress tumor microenvironment, which are increased and activated to prompt immune responses with poor-prognosis solid tumor metastasis [4, 13]. Memory T cells are responsible for long-term immunity and participate in the killing of the antigen when it reenters the body. Effector T cells are directly involved in killing the invasion of foreign antigens and thus play a crucial role in immune function [14]. Naive T cells are considered to be immature and, unlike activated cells, can transform into other types of immune system cells. Programmed death-1 (PD-1) is primarily expressed on activated T and B cells as well as other activated immune cells, which can binds to its major ligand, Programmed death-ligand 1 (PD-L1), and is typically expressed on human cancer cells. When the PD-L1 of cancer cells interacts with PD-1of T cells, the function of T cells is diminished. Moreover, PD-L2 is another ligand of PD-1 and plays similar and opposing roles to that of PD-L1 in T-cell function [15, 16].

In this study, we aimed to disclose the immune microenvironment changes in several immune tissues that accompany tumor growth in mice bearing the same tumor, and then detail the tumor progression changes by immunophenotyping to monitor both changes in the numbers of different lymphoid populations and the expression levels of key molecular players, namely, checkpoints. Furthermore, we double checked the anti-tumor effect of the CTLA-4 antibody treatment by measuring the differences in tumor growth in the same tumor type and then isolated the tumor tissue and characterized it by immunohistochemistry to further validate the immune microenvironment mechanism after therapy [17–19].

## RESULTS

### Different initial CT26 xenograft tumor size leads to differences in anti-tumor efficacy

**Figure 1:**
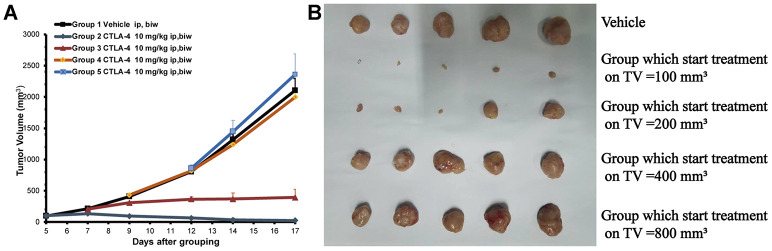
**Different tumor sizes depending on the start treatment (100 to 800 mm^3^) of the subcutaneously inoculated CT26 show the different anti-tumor effects.** The cell numbers of inoculation is 3 × 10^5^ into 100 ul for each mouse. Tumor volume trace after administering anti-CTLA-4 Ab to female BALB/c mice inoculated with the CT26 syngeneic model. (**A**) Volume of tumors from mice inoculated with CT26 subcutaneously. Mean tumor volumes (mm^3^) ± SE (n = 5 mice per group) are shown. Data are statistically significant for anti-CTLA-4 antibody 10 mg/kg intraperitoneal injection, weekly; ***, P < 0.001, on day 17. (**B**) Photos of the tumors isolated from mice inoculated with CT26 cells in the study.

To validate the efficacy of the anti-CTLA-4 (10 mg/kg, biw, ip) treatment on tumors of different sizes in mice subcutaneously inoculated with CT26 cells xenograft, we measure the tumor size after treatment. The tumor volumes were 2,106 ±205mm^3^ on day 17 in the control group treated with vehicle only (Figure 1A). Compared with the control group, the 100 mm^3^ (at start) group treated with the anti-CTLA-4 antibody (10 mg/kg, biw, ip) on day five showed anti-tumor activity with a statistical difference compared with the vehicle group (***P < 0.001) (the tumor volumes were 23±4 mm^3^ vs. 2106±205 mm^3^). The 200 mm^3^ (at start) group start treated with the anti-CTLA-4 antibody (10mg/kg, biw, ip) on day seven produced anti-tumor activity with a statistical difference (**P < 0.01) (the tumor volumes were 394±34 mm^3^ vs. 2,106±205 mm^3^). The 400 mm^3^ (at start) group treated with the anti-CTLA-4 antibody (10mg/kg, biw, ip) on day nine did not produce anti-tumor activity (P > 0.05), as the tumor volumes were 2313±234 mm^3^. The 800 mm^3^ (at start) group treated with the anti-CTLA-4 antibody (10 mg/kg, biw, ip) on day 12 did not show statistically significant differences in anti-tumor activity (P > 0.05), as the tumor volumes were 2648±398 mm^3^. At the end of the study, the tumors were collected and photographed for all of the mice (Figure 1B).

### Tumor cell proliferation was dependent on tumor sizes after treatment with the same concentration of anti-CTLA-4 antibody

**Figure 2:**
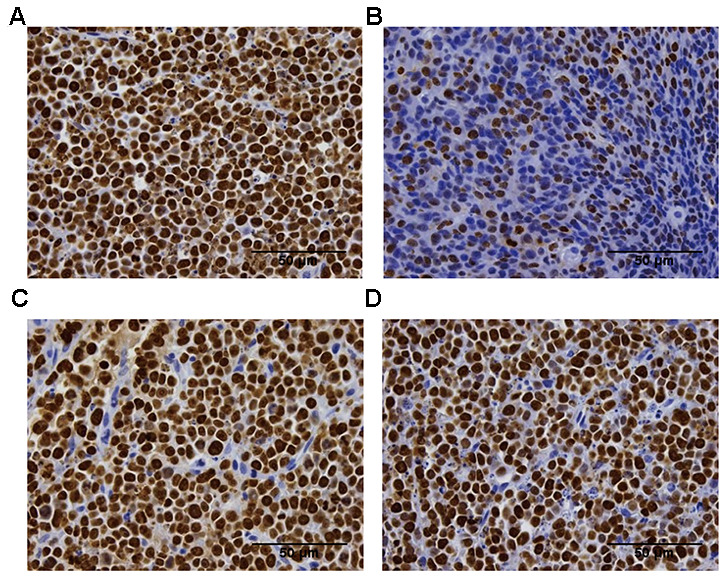
**Ki-67 immunohistochemistry analysis of the proliferation of the tumor tissue in the difference groups.** Ki-67 staining in the vehicle group (**A**), the 200 mm^3^ group (**B**), the 400 mm^3^ group (**C**), and the 800 mm^3^ group (**D**); n = 5 mice per group. Data are statistically significant for anti-CTLA-4 antibody 10 mg/kg intraperitoneal injection weekly; **, P < 0.01 on day17, Photos of the tumors isolated from mice inoculated with the CT26 cells. For group 2 (start treatment with the tumor size of 100mm^3^), no IHC staining is shown due to the small tumor volume on the final day.

To further validate the anti-tumor efficacy of the CTLA-4 antibody on different tumor sizes, we measured the Ki-67 IHC expression in the tumor tissue collected from the in vivo study (Figure 2A). Ki-67 is high expressed means the tumor cells in a state of proliferation. The group which start treatment with 200 mm^3^ showed a different percentage of proliferating cells compared with the vehicle group (23.2% ±5.86 vs. 99.6%±0.79, respectively; **P < 0.01; Figure 2B). The other groups (start treatment with 400 mm^3^ groups and 800 mm^3^ groups) did not show differences compared with the vehicle group (94.4%±2.82 and 97.5%±1.73 vs. vehicle group 99.6%±0.79, respectively; P > 0.05; Figure 2C, 2D).

### Immune cells were characterized by the stage of tumor progression and the immune tissues

**Figure 3:**
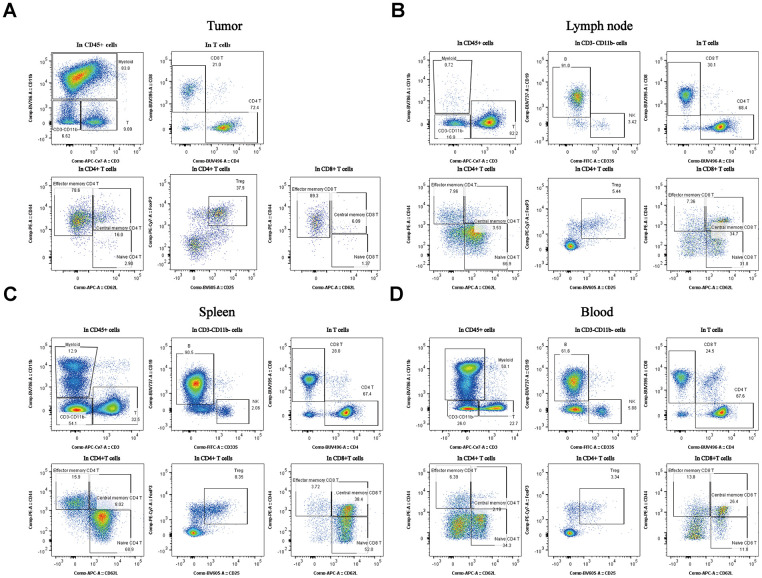
**Percentages of the different subpopulations and expression levels in different tissues of the subcutaneous xenograft model.** Distribution of immune cell subpopulations in the tumor, spleen, lymph node, and blood isolated from the same tumor progression animal model (CT26 cells). The gating schema for the study and the percentage of immune cell subpopulations in TILs (**A**), lymph node (**B**), spleen (**C**), and peripheral blood (**D**).

Myeloid cells were present at much higher percentages in tumors and blood isolated from the mice than in the other tissues (Figure 3A, *P < 0.05). Treg cells were also at much higher percentages in tumors than in the others tissues (Figure 3A, *P < 0.05). The TCM cells were present at much higher percentages in the lymph node than in others tissues (Figure 3B, *P < 0.05). The B cells were present at much higher percentages in the spleen than in others tissues (Figure 3C, *P < 0.05). As for the B cells, the percentages were much higher in the blood samples than in others samples (Figure 3D, *P < 0.05). These data indicate that different immune tissues have different immune subpopulations for a given stage of tumor progression in the same subcutaneous xenograft model. All of the representative figures were from the tumor stage at an approximate tumor volume of 100 mm^3^ in CT26 tumor type.

### The expression levels of immune factors were dependent on tumor sizes and immune tissues

**Figure 4:**
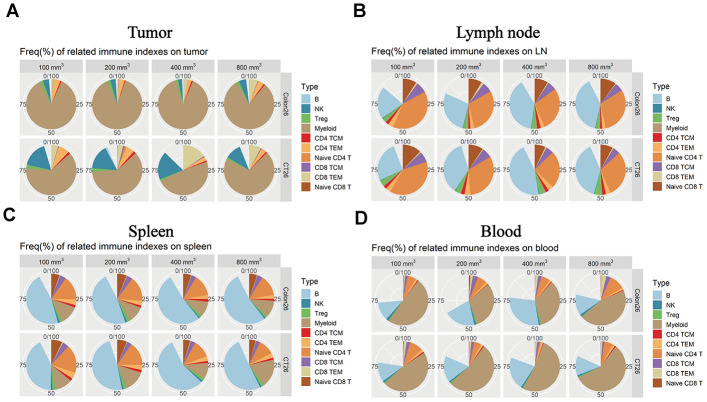
**Immune microenvironment differences accompany the tumor progression changes in the different immune tissues.** The percentage of immune cell subpopulations in different immune tissues isolated from both CT26 and Colon26 animal models in Balb/c female mice. (**A**) Different immune factor expression ratios in TILs. (**B**) Different immune factor expression ratios in the lymph node isolated from the armpit. (**C**) Different immune subpopulations expression ratio in the spleen tissue isolated from an immune-competent mouse. (**D**) Different immune subpopulation expression ratios in peripheral blood isolated from both CT26 and Colone26 animal models.

Immunophenotyping was performed in the different immune tissues for different tumor progression. As for the TILs, the myeloid cells as a substantial ratio of the immune cell types in the two tumor-device models. As is shown, the NK cells and TCM cells in CD4T show differences that correlate with tumor progression (Figure 4A, * P < 0.05). In the lymph node tissue, the B cells and naive CD4T produced different percentages in the all immune factors that correlate with the tumor progression changes (Figure 4B, * P < 0.05). Comparing the animal models (CT26 vs. Colon 26), there were several differences at the expression level for the subpopulations. As for the spleen tissue, the numbers of B cells, naive CD4T, and TEM in CD8T also correlated with differences in the tumor size (Figure 4C, * P < 0.05). The B cells, myeloid, and naive CD4T cells produced a significant difference (Figure 4D, * P < 0.05) accompany with the change of tumor growth stage. Overall, these data suggest that the different tumor growth stages result in different immune environments in the immune tissues.

### Levels of expression of PD-1 and CTLA-4 in each subpopulation correlate with tumor progression in the CT26 xenograft model

**Figure 5:**
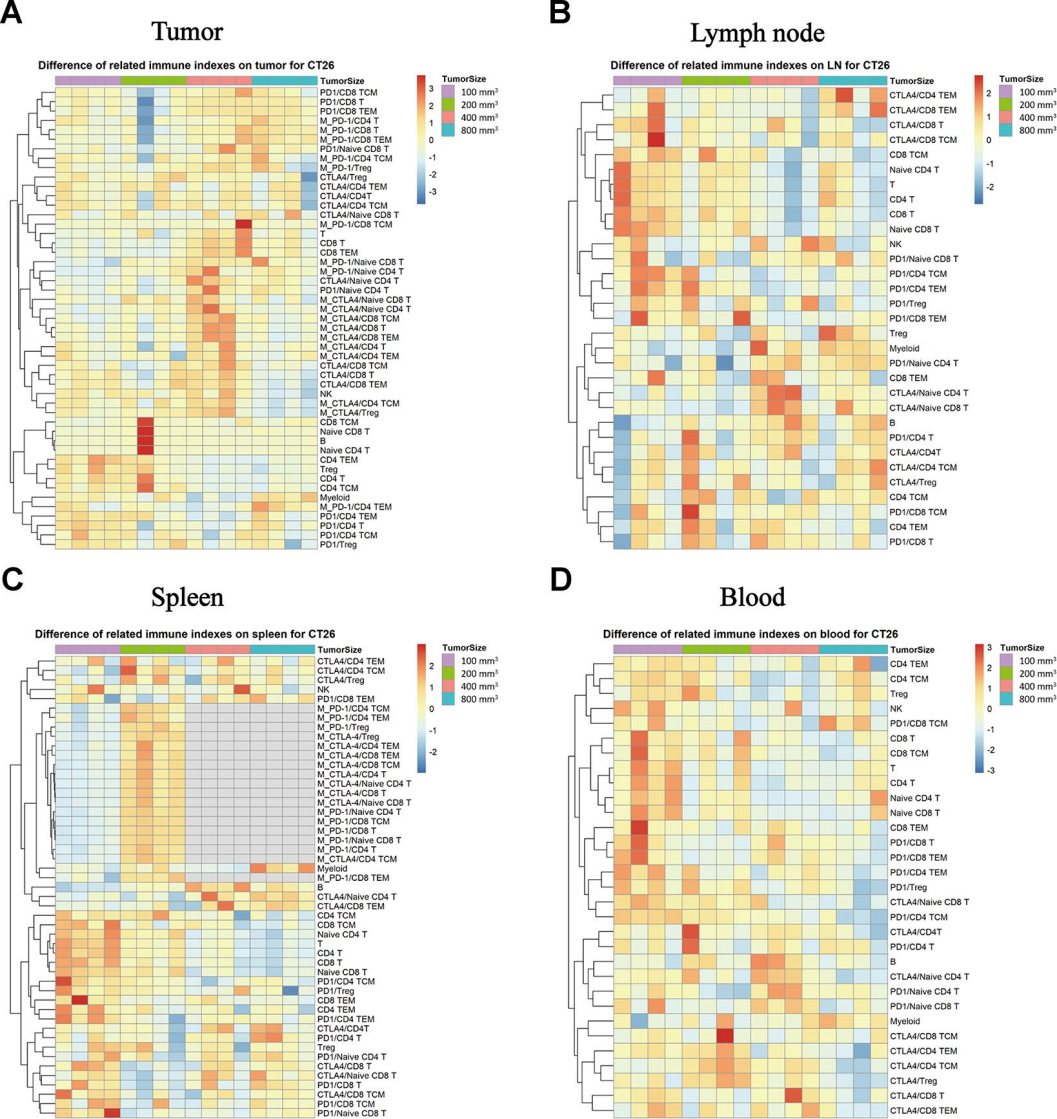
**Expression of PD-1 and CTLA-4 checkpoints in each subpopulation of different immune tissues at different stages of the tumor progression.** The different colors represent the different levels of expression. The differential expression of PD-1 and CTLA-4 in immune cell subpopulations in different immune tissues isolated from the CT26 animal model in Balb/c female mice. (**A**) Different expression levels of PD-1 and CTLA-4 in each subpopulation in TILs. (**B**) Different expression levels of PD-1 and CTLA-4 in each subpopulation in the lymph nodes. (**C**) Different expression levels of PD-1 and CTLA-4 in each subpopulation in the spleen (no sufficient data could be gathered for the MFI of CTLA-4 in the different subpopulations when the tumor size was 400 mm^3^ and 800 mm^3^). (**D**) Different expression levels of PD-1 and CTLA-4 in each subpopulation in the peripheral blood samples in the CT26 animal model. The statistical analysis in this figure was obtained by analyzing the raw data. The dendrogram was plotted from the raw data of each subpopulation and biomarker using the R language.

We evaluated whether the immune checkpoint factors (PD-1 and CTLA-4) expression in the difference immune subpopulations changes with tumor growth progression. In the TILs, the PD-1 and CTLA-4 expression increased with tumor growth, and there were much higher levels when the tumor sizes were about 400 mm^3^ in each subpopulation (Figure 5A, * P < 0.05). In the lymph node tissue, there was an opposing pattern, as the CTLA-4 and PD-1 expression levels were higher when the tumor sizes were 100 mm^3^ than in the other tumor growth stages (Figure 5B, * P < 0.05). In the spleen tissue, the expression level of the PD-1 and CTLA-4 in each subpopulation was the same as in the lymph node, the group which start treatment with 100 mm^3^ produce a significant difference compare to the other groups (Figure 5C, * P < 0.05). In the peripheral blood, the expression levels were the same as in the lymph node tissue with the tumor size was in 100 mm^3^, and there was a much higher level of both PD-1 and CTLA-4 in the small tumor sizes (such as 100 mm^3^) than in the big ones (Figure 5D, * P < 0.05).

### Expression levels of PD-1 and CTLA-4 in each subpopulation correlate with the tumor progression in the Colon26 xenograft model

**Figure 6:**
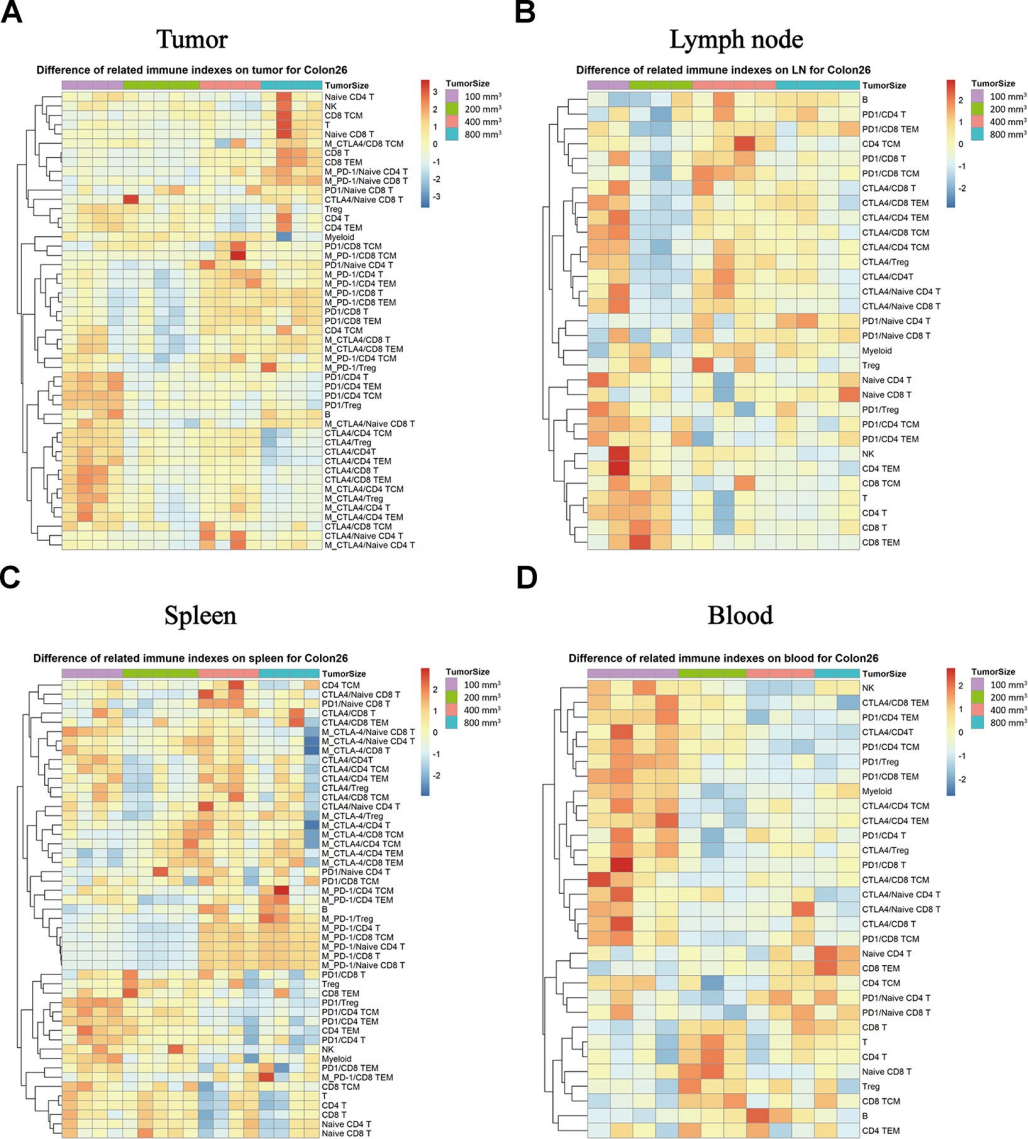
**Expression of the PD-1 and CTLA-4 checkpoints in each subpopulation of different immune tissues at each stage of tumor progression.** The different colors represent different levels of expression. The differential expression of PD-1 and CTLA-4 in immune cell subpopulations in different immune tissues isolated from the Colon26 animal model. Different expression levels of PD-1 and CTLA-4 in each subpopulation in TILs (**A**), lymph nodes (**B**), spleen (**C**), and peripheral blood samples (**D**) in the Colon26 animal model. The statistical analysis in this figure was obtained by analyzing the raw data. The dendrogram was plotted from the raw data of each subpopulation and biomarker using the R language.

We monitored the expression of the PD-1 and CTLA-4immune checkpoints factors in the different immune subpopulations of the immune tissues as a function of tumor progression. In TILs, the CD8T, TEM in CD8T, NK and other subpopulations exhibited increasing expression percentage that correlated with the tumor progression changes, but the PD-1 and CTLA-4 expression intensity correlated negatively with the tumor growth stage (Figure 6A, * P < 0.05) in the Colon26 tumor animal model in both the tumor and the lymph node tissue (Figure 6B, * P < 0.05). In the spleen tissue, the expression intensity of the PD-1 increased with the tumor growth, unlike the expression of CTLA-4 in each subpopulation, which decreased with tumor growth (Figure 6C, * P < 0.05). In the peripheral blood, the percentage was the same for each subpopulation, and the levels were much higher for the tumor sizes at 100 mm^3^ (a start) than for the others in the Colon26 animal model (Figure 6D, * P < 0.05).

### Differences in the ratio of CD4+ Effector T/regulatory T cells (Teff/Treg) and CD8 + T/Treg (T/regulatory T cells) in each immune tissue accompanied the tumor progression changes in the CT26 and Colon26 xenograft model

**Figure 7:**
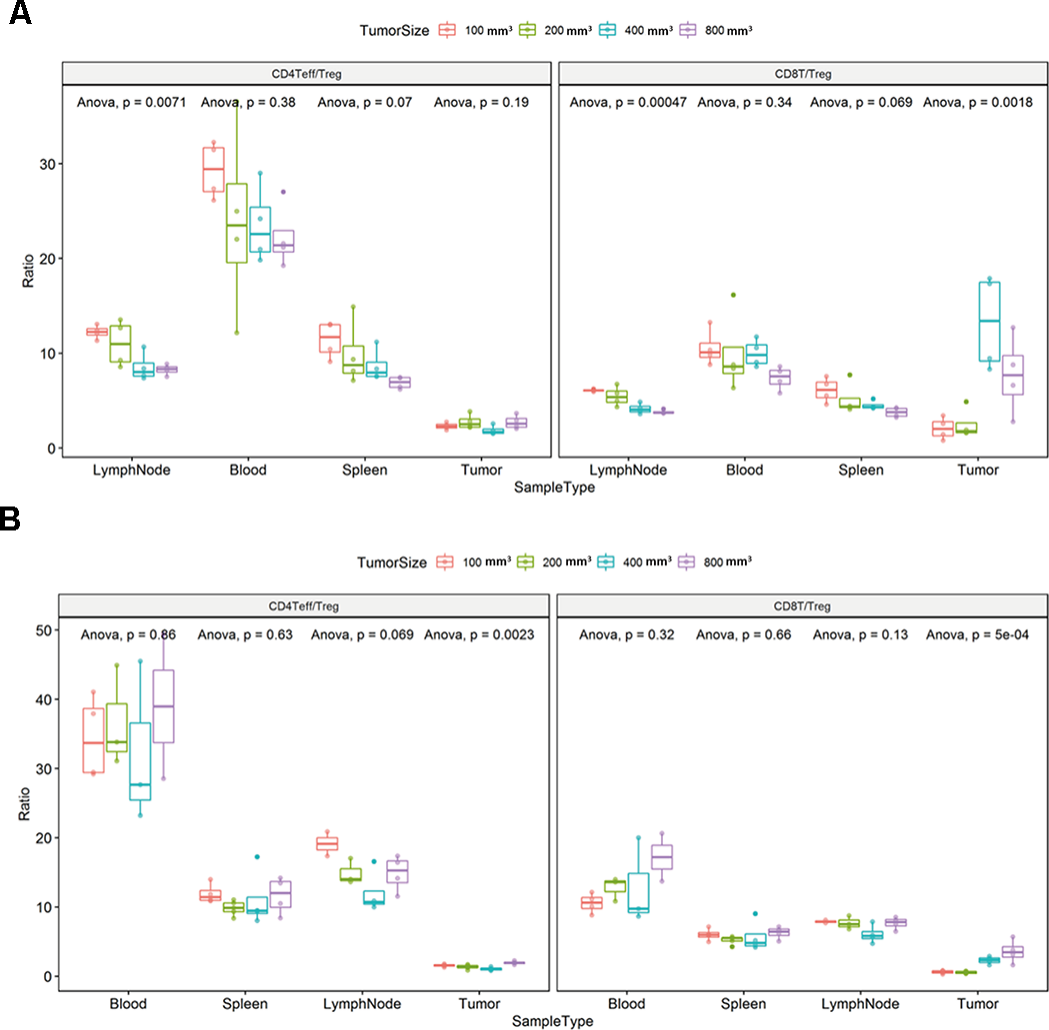
**Ratio of CD4+ Effector T / regulatory T cells (Teff/Treg) and CD8+ T/Treg (T/regulatory T cells) in each immune tissue.** The error bar represents the variation of the data in the same group. The different colors represent the different tumor sizes in the corresponding tissues. (**A**) Differential ratio of Teff/Treg in CD4T and CD8T/Treg in the difference tumor progression phase in the CT26 animal model. (**B**) Differential ratio of Teff/Treg in CD4T and CD8T/Treg in the difference tumor progression phase in the Colon26 animal model. The statistical analysis in this figure was obtained by analyzing the raw data.

We found that the ratio of CD4Teff/Treg and CD8T/Treg in the lymph node tissue changed with the tumor progression (Figure 7A, ** P < 0.001). The ratio of CD8T/Treg in the tumor tissue also changed with the tumor progression in the CT26 animal model (Figure 7A; * P < 0.05). Likewise, the ratio of CD4Teff/Treg and CD8T/Treg in the tumor tissue changed with the different tumor growth phase (Figure 7B, * P < 0.05).

## DISCUSSION

CT26 and Colon26 tumor cell lines are colon carcinoma lines used in pharmacodynamic experiments involving immune-related checkpoint antibodies and related immune mechanisms. Female Balb/c mice were inoculated with CT26 cells in the right upper flank. The isolated tumors were evaluated for changes in tumor volume following treatment with anti-CTLA-4, changes in immune cell subpopulations, and the expression of critical immune checkpoints by flow cytometry. Meanwhile, a pathological assay was performed on the tumors isolated from the mice.

We detected each lymphocyte or myeloid subpopulation in the spleen, blood, tumor, and lymph node at different tumor phases, and monitored the treatment with the same antibody to evaluate how the treatment efficacy varies with the initial tumor size. Furthermore, we obtained pathology results for validation.

The immune microenvironment changes with the different tumor growth phases [20–22]. In this study, we further subdivided the lymphocyte subsets and compared the expression of immune cells in different immune tissues of the mouse models, such as the spleen, peripheral blood, and lymph nodes, besides the tumors. Furthermore, we analyzed the expression of immune checkpoint factors in each subpopulation, namely CTLA-4 (cytotoxic T-lymphocyte-associated protein 4) and PD-1(Programmed cell death protein 1) [9, 23].

CTLA4 or CTLA-4 (cytotoxic T-lymphocyte-associated protein 4), also known as CD152 (cluster of differentiation 152), is a protein receptor that represses the immune response. Thus, we focused on the expression differences in each subpopulation and disclosed the different levels in different immune tissues of the tumor-bearing mouse model. As for the PD-1 (Programmed cell death protein) immune checkpoint factor, it is known to inhibit autoimmunity through two mechanisms: the promotion of apoptosis of antigen-specific T cells in lymph nodes and the reduction of apoptosis in regulatory T cells [24–26]. Combining the function characteristics of the two factors, their expression level in related subpopulations in different immune tissues, and the differential ratio of Teff/Treg in CD4T and CD8T/Treg in the different immune tissue produced statistically significant differences [27, 28]. It could be adequately demonstrated that the changes in immune microenvironment also correlate with different tumor sizes accompany with tumor growth and tumor the micro level of progression changes [16, 29, 30]. The results of the in vivo efficacy experiment and the tumor tissue analysis Ki-67 IHC could clearly support this conclusion [31–33].

As stated in previous articles, subcutaneous mouse models using different inoculation sites also led to differences in anti-tumor efficacy and changes in the immune environment [34]. Therefore, it can also reflect the substantial differences in the immune microenvironment and responses to relevant anti-tumor drugs that are correlated with the tumor growth phase changes. The above conclusions can be clearly drawn from the results of the pathological analysis and efficacy results of tumor treatment with the same concentration of antibody at different tumor growth stages. The main relevance of our study was to show that different tumor progression phases not only result in differences in anti-tumor efficacy, but also induce differences in the immune response and immune microenvironment, even when the tumor type is the same [35].

In this study, we evaluated the immune microenvironment changes during tumor progression by immunophenotyping and biomarker analysis of tumors in different growth phases and the corresponding immune organs, namely the spleen, peripheral blood, and lymph nodes, using immunohistochemistry and flow cytometry. Furthermore, we disclosed that there are differences in the drug response, pathological features, and immunophenotyping depending on the tumor size, even for tumors of the same type. In addition, we describe the phenotyping of multi-type subpopulations and level of expression of biomarkers for better illustration of the change in the immune microenvironment that regulates and influences the process of tumor growth in colon cancer [36]. Our study reveals that different tumor growth phases correspond to different immune states. What we found may be useful to detail further the changes in the immune microenvironment and the different responses to clinical medication, and ultimately to guide the treatment of cancer patients. Taking into consideration that all different *in vivo* efficacy and tumor-immune phenotyping results, the higher expression levels of CTLA-4 in phenotyping were associated with more effective treatment of the anti-CTLA-4 antibody, implying that the effect of immunotherapy is mainly dominated by tumor-immune microenvironment with depending on tumor growth phases regardless of tumor type.

Our work has fully demonstrated the differences in the expression levels of immunophenotypes and biomarkers in different immune organs that occur during tumor proliferation in the colorectal cancer. One downside is that we are only disclosing the immune microenvironment in one type of tumor. In the future, the changes in the immune microenvironment of various tumor types need to be further clarified, including breast cancer, gastric carcinoma, and lung cancer. This study is a significant contribution for fundamental research that may be relevant for the immunotherapy of solid tumors in the near future.

## MATERIALS AND METHODS

### Cell culture

CT26 and Colon26 colon carcinoma cells with high metastatic potential were obtained from the American Type Culture Collection (ATCC; ATCC-CRC-2638, ATCC-CRC-2534). The cells were maintained in Roswell Park Memorial Institute (RPMI) 1640 culture medium (Gibco BRL, Rockville, MD, USA) supplemented with 10% fetal bovine serum (FBS, Life Technologies, Carlsbad, CA, USA) and 100 U/mL penicillin at 37°C in a humidified atmosphere containing 5% CO_2_.

### Antibodies and reagents

The following reagents were used in the flow experiments: CD45 (BD, 560510), CD8 (BD, 565968), CD4 (BD, 564667), CD19 (BD, 564296), Live/Dead (Invitrogen, L34964), CD25 (BD, 563061), PD-1 (BioLegend,135231), Isotype (BioLegend,400551), CD11b (BioLegend,101243), CD335 (BD,560756), CTLA-4 (BioLegend,106316), Isotype for CTLA-4 (BioLegend,400932), CD44 (BioLegend,103007), Foxp3 (eBioscience, 25-5773-82), CD62L (BioLegend,104412), and CD3 (BD,560590); 10 × Red Blood Cell Lysis Solution (BD,342123), DPBS (Corning-21-031-CV), Staining buffer (eBioscience-00-4222), RPMI 1640 medium (Gibco-22400-089), Foxp3 / Transcription Factor Staining Buffer Set (eBioscience-00-5523), Purified Rat Anti-Mouse CD16/CD32 (Mouse BD Fc Block™) (BD-553142), and Fixation Buffer (BD-554655). The following reagents were used in the IHC experiments: Xylene (SCR,10023418), 75% Ethanol (SCR,801769610), 95% Ethanol (GENERAL-REAGENT, G73537D), Ethanol, Absolution (GENERAL-REAGENT, G73537G), SignalStain® Citrate Unmasking Solution (10X)(MAIXIN, B548117-0500), Hydrogen Peroxide Solution, 30% (SCR, 10011208), Goat Serum, New Zealand Origin (Thermo Fisher, 16210072), EnVision FLEX WASH BUFFER (20X)(DAKO, K8000), Antibody Diluent (DAKO, S2022), Ki-67 (D2H10) (CST,9027), Rabbit (DA1E) mAb IgG XP® Isotype Control (CST,3900), EnVision + System-HRP Labelled Polymer Anti-Rabbit (DAKO, K4003), DAB Detection system (Maixin, DAB-0031), Haematoxylin (Harris) (Baso, BA-4041), Neutral Balsam (Huashen, ZLI-9610), Ultra-Thin Section Blade (FEATHER, R35), Superfrost Plus Microscope Slides (Fisher, 12-550-15), and Microscope Cover Glass (CITOTEST, 10212450C).

### Mice

All of the procedures related to animal handling, care, and treatment were performed following the guidelines approved by the institutional committee for animal welfare of the South China University of Technology.

### Anti-CTLA-4 treatment methods

Anti-CTLA-4 (BioX cell, 616116J213) was formulated to the stock concentration of 10 mg/mL for further therapeutic dosages, and then the treatments were initiated with the desired dosages when the average tumor reached 100 mm^3^, 200 mm^3^, 400 mm^3^, and 800 mm^3^. Treatments were administered twice a week by intraperitoneal injection.

### Experimental protocol

To make the CT26 model, 8-week-old female Balb/c mice (purchased from Lingchang Biology, LTD Co.) were inoculated with 3 × 10^5^ tumor cells in the right upper flank. The volume of each tumor was acquired using the formula V = 0.5*(A*B^2^), where A and B are the longer and shorter diameters of the tumor, respectively, and are measured using calipers. As for the CT26 model, the other mice were separated into groups by tumor volume before treatment with anti-CTLA-4, intraperitoneal, bi-weekly when the mean tumor sizes reached to 100 mm^3^, 200 mm^3^, 400 mm^3^, and 800 mm^3^. The tumor volume for each time point was recorded, and, when the average tumor volumes reached more than 2,000 mm^3^ in the vehicle group, the mouse was sacrificed, and the tumor was collected for IHC analysis.

### Flow cytometry

The CT26 and Colon26 colon carcinoma cell lines were inoculated into the 8-week-old female Balb/c mice, as described above. Mice were inoculated on the right upper flank. The animals were sacrificed when the mean target tumor volumes reached 100 mm^3^, 200 mm^3^, 400 mm^3^, and 800 mm^3^, and we collected the blood, spleen, tumor tissue, and lymph node from each mouse. TILs were harvested from fresh tumors using the following methods: (1) tumors were digested with hyaluronidase, collagenase, and deoxyribonuclease in tubes; (2) following a wash, the tumor cells were diluted to a density of 1 × 10^7^ cells/mL; for the spleen: the organ was ground on a 70 μm cell strainer with a 2 mL syringe and washed with DPBS to collect the cells into a tube; the cells were centrifuged at room temperature, the supernatant was removed, and the cells were resuspended with 10 mL 1 × Red Blood Cell Lysis Solution for 1 min at room temperature; the incubation was terminated, and the sample was centrifuged at room temperature, and then the cells were resuspended to a density of 1 × 10^7^ cells/mL; for the blood samples: anticoagulant whole blood was transferred into a 15 mL centrifuge tube; cells were lysed with 1 × Red Blood Cell Lysis Solution to make a 1:19 dilution; lysis was terminated, the sample was centrifuged at room temperature, and then the cells were resuspended at a density of 1 × 10^7^ cells/mL; for the lymph node samples: the tissue was gently laid flat on the slide, and then the cells were continuously eluted with PBS, centrifuged, and resuspended at a density of 1 × 10^7^ cells/mL. The staining procedure for flow was as follows: 1 × 10^6^ cells were resuspended in 100 μL staining buffer composed of PBS and incubated with 1 μL purified rat Anti-Mouse CD16/CD32 for 5 min in the dark at 4°C. Then 1 μL of fluorescently conjugated antibody mixture was added to the cell suspensions, and these were incubated for 30 min in the dark at 4°C. After extracellular staining, the cells were suspended with 100 μL Fixation/Permeabilization buffer, incubated at 4°C for 30 min in the dark, and then resuspended with 200 μL 1 x Permeabilization Buffer per well. The cells were centrifuged, and 100 μL of 1 x Permeabilization Buffer and intracellular markers were added into each well. The cells were incubated at 4°C for 30 min in the dark, and then centrifuged and washed twice. The stained cells were washed twice again and resuspended in 300 μL staining buffer. FACS analyses were performed within 24 h.

### IHC analysis

The slides were deparaffinized and rehydrated (Xylene: 5 min, three times; 100% alcohol: 5 min, twice; 95% alcohol: 3 min; 75% alcohol: 2 min; dH_2_O: 5 min). Antigen retrieval. (Antigen retrieval solution preparation). The citric acid powder (Thermofisher, Cat#: TL-125-HL) was dissolved with distilled water into a 10X standby solution. For endogenous peroxidase quenching, each slide was treated with 200 μL 3% H_2_O_2_ for 5 min at RT and then rinsed for 3 min with 1x wash buffer for three times. For protein blocking, the slides were treated with 200 μL 5% normal goat serum. After primary antibody and secondary antibody incubation, for color development, 200 μL of a working solution of DAB was added, and for counterstaining the slides were treated with Hematoxylin, differentiated with HCl-Alcohol, and then dehydrated. Coverslips were mounted onto the slides. Ki-67 score was analyzed using Aperio Nuclear V9 algorithm (Version. 9.1).

### Statistical analysis

GraphPad Prism Software was used for statistical analyses. The analysis was performed with an unpaired Student's t-test and two-way ANOVA test. Data are presented as means ± SEM of three independent experiments. Values of P < 0.05 were considered to be statistically significant.
